# Unveiling the Impact of Total Body Fluid Dynamics on Chronic Rhinosinusitis With Sinonasal Polyposis

**DOI:** 10.7759/cureus.78916

**Published:** 2025-02-12

**Authors:** Richi Sinha, Amit Abhinandan, Mani Mala, Rakesh K Singh

**Affiliations:** 1 Otolaryngology - Head and Neck Surgery, Indira Gandhi Institute of Medical Sciences, Patna, IND

**Keywords:** chronic rhinosinusitis, fluid dynamics, pathogenesis, regression analysis, sinonasal polyposis, total body water

## Abstract

Background: This study investigated the role of total body fluid dynamics in developing polyposis in patients with chronic rhinosinusitis. There is a significant gap in our understanding of body fluid dynamics in the pathogenesis of sinonasal polyposis. This preliminary research will be sensitized to explore hidden facts in this field further.

Methods: In this cross-sectional study at a tertiary care hospital, 60 participants were enrolled, comprising 30 patients diagnosed with sinonasal polyposis and 30 controls without polyps. The measured parameters included total body water, excess body water, serum osmolality, albumin, creatinine, sodium, eosinophil count, arterial pH, and hematocrit. The t-test/Mann-Whitney U test and chi-square test were used for continuous and categorical variable analysis, respectively. Univariate and multivariate logistic regression was done to identify potential predictors, and model performance was evaluated using the receiver operating characteristic (ROC) curves, with an optimal classification cut-off of 0.5 for the area under the curve (AUC).

Results: The mean age of cases was 34.03 ± 12.76 years, and that of controls was 34.77 ± 12.06 years. Amongst cases, 60% were males and 40% were females, while the control group had an equal distribution of males and females. The mean total body water level in patients with sinonasal polyposis was slightly higher than in those without polyps (34.42 ± 5.26 l vs. 32.38 ± 5.86 l). Moreover, the likelihood of developing sinonasal polyposis was twice as high in patients with excess body water compared to those without, as suggested by an adjusted odds ratio (OR) of 2.08. However, this association was not statistically significant (95% CI: 0.83-5.19, p = 0.116). Patients with polyposis also showed higher serum creatinine levels (0.94 mg/dL) than controls (0.80 mg/dL, p = 0.002). Univariate logistic regression indicated that a 1 mg/dL increase in serum creatinine level was associated with a 2.4-fold increase in the likelihood of polyposis (OR = 2.42, 95% CI: 1.30-4.50, p = 0.005). Multivariate analysis confirmed the significance of serum creatinine level (adjusted OR = 3.36, 95% CI: 1.42-7.97, p = 0.006), with an AUC of 0.803, demonstrating good discriminative ability. The model's accuracy, sensitivity, and specificity were 66.7%, 73.3%, and 60%, respectively.

Conclusions: Significantly elevated serum creatinine levels in patients with sinonasal polyposis may suggest a potential link with renal function or fluid regulation, warranting further research in larger cohorts to clarify this relationship. The regression model indicated a positive correlation between excess body water and the development of polyposis, with a weaker trend observed for other parameters. However, due to insufficient evidence, further research involving larger sample sizes across diverse geographic regions is recommended to validate their roles in the pathogenesis of sinonasal polyps.

## Introduction

Chronic rhinosinusitis (CRS) affects 10% of the global population. It has been traditionally classified into two types depending on the presence of polyps: CRS with nasal polyps (CRSwNP) and CRS without nasal polyps (CRSsNP) [1]. Several factors have been linked to the development of polyps, including geographical and racial variations, environmental exposures, imbalances in sinus microbiota, mucociliary clearance dysfunction, epithelial barrier impairment, and immune response disruption [2,3]. Polyps are pale and edematous masses, mainly originating in the middle meatus [1,4]. They are predominantly composed of water and thus follow the hypothesis that the key to the pathogenesis of nasal polyps may lie around the factors contributing to excess water flow through the nasal epithelium [5]. We explored total body fluid dynamics and its role as a likely candidate in developing this disease.

Previous research on the sinonasal epithelium has identified differences in aquaporin types between diseased and healthy individuals and lower serum albumin concentrations in patients with nasal polyps [5-7]. Local inflammation resulting from sinonasal epithelial damage has also been implicated [8]. However, based on a complete literature review, the role of broader body fluid dynamics such as total body water (TBW), electrolytes, and pH in this disease remains largely unexplored. This is a poorly researched area and the results could have far-reaching implications for the current management of this condition. This could herald a new hope for patients who derive partial or no benefit from topical and systemic corticosteroids, which currently form the cornerstone of CRS treatment.

TBW can be estimated from anthropometric measurements using various equations, of which Watson’s formula provides the most accurate measurement [9]. When we look at the architecture of sinonasal polyps, we find that they are formed by fluid influx into the lamina propria of the sinonasal Schneiderian membrane [6,10].

The main goal of this study was to determine whether there is a correlation between sinonasal polyposis and increased body water levels. Secondary objectives included investigating potential associations between sinonasal polyposis and other biochemical markers like serum albumin, serum creatinine, serum calcium, hematocrits, serum sodium, pH, and eosinophil count.

## Materials and methods

Study design and setting

This prospective, cross-sectional, observational study examined the relationship between body fluid dynamics and sinonasal polyposis at a single tertiary care teaching hospital between July 2022 and April 2024. The study was approved by the Institutional Ethics Committee, Indira Gandhi Institute of Medical Sciences, Patna, India (approval number: 601/IEC/IGIMS/2022; dated: 18/07/2022) and conformed to the recognized standards of the World Medical Association Declaration of Helsinki.

Study participants

The inclusion criteria were adult patients aged 18 to 60 years with clinically and radiologically proven sinonasal polyposis in cases and clinically and radiologically negative sinonasal polyposis in controls who provided written informed consent to participate in the study. The presence of polyps on nasal endoscopy with two cardinal symptoms (progressive nasal obstruction, nasal and/or facial congestion, rhinorrhea, and hyposmia or anosmia) confirmed the clinical diagnosis. Radiological diagnosis was based on a CT scan that showed hypodense partial or complete opacification of the nasal cavity or the paranasal sinuses with or without enlargement of infundibula and gas-fluid levels. Patients were excluded from the study if they were under 18 or over 60 years of age or had been diagnosed with any comorbidities such as diabetes, hypertension, renal disease, liver disease, thyroid or other hormonal disorders, or neurological, musculoskeletal, or autoimmune disorders. Renal disease was defined using standard laboratory cutoffs: serum creatinine levels >1.3 mg/dL in males and >1.1 mg/dL in females or serum albumin levels <3.5 g/dL. Participants were also excluded if they were pregnant or had other benign or malignant sinonasal or nasopharyngeal conditions. Additionally, patients with a history of smoking, tobacco use, alcohol intake, or any other substance abuse, as well as those taking any topical or systemic medication, were excluded.

Sample size and sampling

The sample size for this study was calculated based on a previous study that reported a 4% prevalence of sinonasal polyposis in the Indian population [2] and a power calculation. To detect differences in TBW between the two groups with 80% power and a significance level of 0.05, 60 participants with a cases-to-control ratio of 1:1 were required. Cases were identified from the patients presenting to the otorhinolaryngology department with sinonasal complaints. For each case, we recruited an age and sex-matched control from the same setting who presented with other complaints (e.g., ear, throat, or neck issues) but had no sinonasal complaints.

Data collection

The primary outcome variables were TBW and excess body water, and the secondary variables were serum albumin, serum creatinine, serum calcium, hematocrits, serum sodium, arterial pH, and eosinophil count. The covariates included were age, sex, weight, and height. Demographic details such as name, age, and sex were noted. A detailed history was obtained. Anterior rhinoscopy and rigid diagnostic endoscopy were performed. Plain and contrast CT of the nose and paranasal sinuses were done. Sinonasal polyposis was diagnosed based on clinical and radiological findings. The height and weight of all participants were recorded using the same instrument. The TBW in both groups was estimated using Watson’s formula [9], using their height, weight, and age since this formula has established accuracy and widespread use in clinical and research settings. Compared to alternative methods such as bioelectrical impedance analysis or isotope dilution, Watson’s formula provides a simple, validated approach based on anthropometric parameters, making it a practical choice for large-scale studies. No formal calibration or external validation of Watson’s formula was conducted within this study; however, its reliability has been demonstrated in prior research, and it was applied uniformly across all participants. To minimize variability, sample collection times were standardized, and participants were assessed for adequate hydration by evaluating skin turgor and vital signs before collection. The ideal body weight of all participants was then estimated using the Devine formula [11], from which the ideal body water was calculated using the same method. Excess body water was then calculated by subtracting the ideal body water from the TBW. Participants' complete blood count, liver function, and kidney function tests were also taken. Serum osmolality was estimated using the following formula: serum osmolality = 2 x Na+ + glucose / 18 + blood urea nitrogen / 2.8). Eosinophil count, serum albumin, serum creatinine, serum sodium, serum calcium, hematocrit value, and arterial pH were noted down. The biochemical tests were conducted using the Nova Biomedical Stat Profile pHOx Ultra analyzer (Nova Biomedical, Waltham, MA, USA) and for albumin and creatinine measurements using the Beckman Coulter AU 800 automated chemistry analyzer (Beckman Coulter, Brea, CA, USA). All analyses were performed following the manufacturer's standard protocols, including routine calibration and internal quality control procedures to ensure the accuracy and reliability of the results.

Statistical analysis

All statistical analyses were conducted using Microsoft Excel (v16.88; Microsoft Corporation, Redmond, WA) with the Analysis ToolPak add-in and Realstats-Mac-2011 add-in to evaluate predictors associated with sinonasal polyposis. Descriptive statistics were computed for each predictor variable, including the mean, standard deviation, median, interquartile range (IQR), minimum and maximum values, skewness, and kurtosis. Normality was evaluated using the Shapiro-Wilk test to determine appropriate statistical tests. After confirming equal variances with an F-test, a t-test (two samples assuming equal variances) was used to compare continuous variables (weight, height, serum albumin, serum creatinine, serum sodium, and hematocrit). The Mann-Whitney U test was used for continuous variables that did not meet the assumptions of the t-test (age, TBW, excess body water, serum osmolality, eosinophil count, serum calcium, and arterial pH). The chi-square test assessed the association between the categorical variable (sex) and sinonasal polyposis. Predictor variables were evaluated using univariate and multivariate logistic regression models after they were standardized by converting them to z-scores. Further, those variables with variance inflation factor (VIF) values above five were removed from the final model to avoid multicollinearity issues. The performance of the final multivariable logistic regression model and each univariate model was assessed using the receiver operating characteristic (ROC) curves. The area under the curve (AUC) was calculated to evaluate the discriminative ability of each model. Models with an AUC closer to 1.0 were considered to have a better discriminative ability. The optimal cut-off for classification was set to 0.5.

## Results

**Figure 1:**
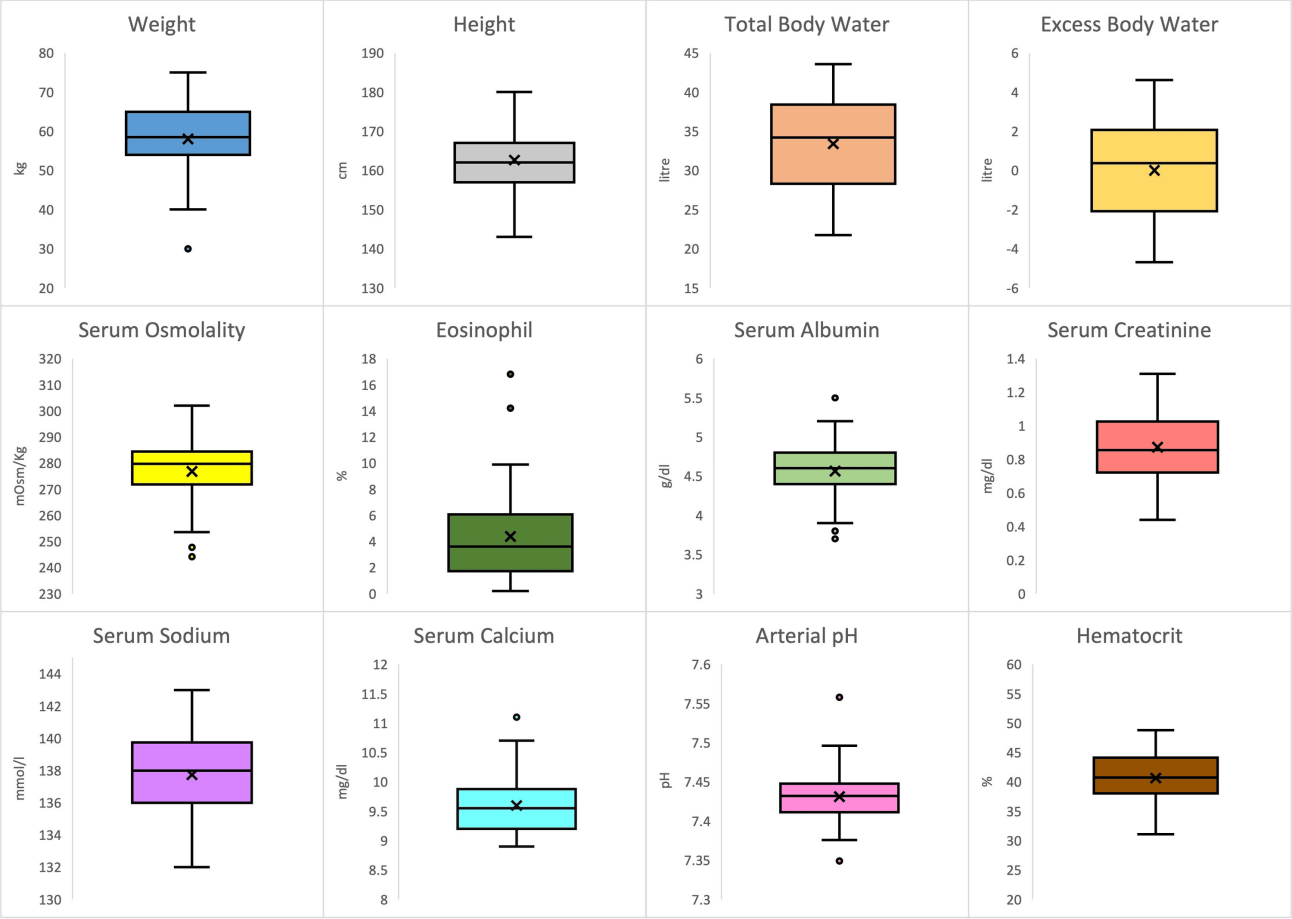
A boxplot of the distribution of the predictor variables in patients with sinonasal polyposis. The figure illustrates the data's central tendency (median) and spread (interquartile range), with whiskers indicating variability outside the upper and lower quartiles. The dots represent outliers in the data for each variable.

Sixty subjects (30 cases and 30 controls) were recruited to examine the relationship between body fluid dynamics and sinonasal polyposis. The mean age of cases was 34.03 ± 12.76 years, and that of controls was 34.77 ± 12.06 years, with a range of 18-60 years for both groups. The sex distribution amongst cases was 18 (60%) males and 12 (40%) females, while the control group had an equal distribution of males and females. No significant difference was observed in the age and sex distribution between the two groups. The mean values of all the predictor variables and covariates between the two groups are summarized in Table 1, and their distributions are shown in the box plot in Figure 1.

**Table 1:** Descriptive data of study participants and comparison of predictor variables between patients with and without sinonasal polyposis. A t-test was used to compare weight, height, serum albumin, serum creatinine, serum sodium, and hematocrit, while the Mann-Whitney U test for two independent samples was used to compare age, total body water, excess body water, serum osmolality, eosinophil count, serum calcium, and arterial pH. The chi-square test was used to compare genders. * P < 0.05 was considered statistically significant.

Description	With sinonasal polyposis (N = 30)	Without sinonasal polyposis (N = 30)	p-value
Age (years), mean ± SD	34.03 ± 12.76	34.77 ± 12.06	0.752
Males, n (%)	18 (60%)	15 (50%)	0.436
Weight (kg), mean ± SD	59.93 ± 7.45	56.10 ± 9.80	0.093
Height (cm), mean ± SD	164.12 ± 7.51	161.20 ± 8.76	0.171
Total body water (l), mean ± SD	34.42 ± 5.26	32.38 ± 5.86	0.159
Excess body water (l), mean ± SD	0.15 ± 2.54	-0.14 ± 2.65	0.513
Serum osmolality (mOsm/kg), mean ± SD	274.31 ± 13.65	279.35 ± 9.92	0.142
Eosinophil (%), mean ± SD	4.23 ± 3.38	4.52 ± 3.91	0.994
Serum albumin (g/dl), mean ± SD	4.58 ± 0.30	4.55 ± 0.38	0.737
Serum creatinine (mg/dl), mean ± SD	0.94 ± 0.17	0.80 ± 0.18	0.002*
Serum calcium (mg/dl), mean ± SD	9.58 ± 0.36	9.62 ± 0.49	0.947
Serum sodium (mmol/l), mean ± SD	137.57 ± 2.42	137.90 ± 2.37	0.591
Hematocrit (%), mean ± SD	40.50 ± 3.76	40.82 ± 4.39	0.767
Arterial pH, mean ± SD	7.43 ± 0.03	7.43 ± 0.03	0.947

The mean TBW level of patients with sinonasal polyposis (34.42 ± 5.26 l) was higher than those without polyposis (32.38 ± 5.86 l), and the mean excess body water of cases (0.15 ± 2.54 l) was more than controls (-0.14 ± 2.65 l). However, this difference was statistically insignificant. Among secondary predictor variables, serum creatinine levels were significantly higher in patients with polyposis (0.94 mg/dL) than those without (0.80 mg/dL), with a p-value of 0.002. Weight and serum osmolality emerged as the next most noteworthy predictors, with p-values of 0.09 and 0.14, respectively. Specifically, patients with polyposis tended to have higher body weight and lower serum osmolality than controls. We also noted slightly higher serum albumin levels in cases than in controls (4.58 ± 0.30 g/dl and 4.55 ± 0.38 g/dl, respectively), while the levels of all other biochemical markers (eosinophil count, serum calcium, serum sodium, and hematocrit) were found slightly lower in cases, though these differences were statistically insignificant. The arterial pH of both groups was found to be similar (7.43 ± 0.03) (Table 1).

Univariate logistic regression analysis revealed that while there was a positive association between body water levels and sinonasal polyposis, this association did not reach statistical significance. The odds ratio (OR) indicated a potential increase in odds with higher body water levels, but the wide confidence interval undermines the strength and significance of this relationship. Serum creatinine was identified as a significant predictor, with a 95% confidence interval (CI) of 1.30-4.50 and a p-value of 0.005. For every 1 mg/dL increase in serum creatinine, the odds of having sinonasal polyposis increased by approximately 2.4 times. No other secondary variables reached statistical significance in the univariate logistic regression analysis, and the OR was close to 1, except for serum osmolality. It was noted that higher serum osmolality decreases the likelihood of sinonasal polyposis as reflected by an OR of 0.641 and a coefficient of -0.412 (Table 2).

**Table 2:** Logistic regression analysis of predictors for sinonasal polyposis. The table shows the unadjusted and adjusted odds ratio (OR) and confidence interval (CI). The overall multivariate model is statistically significant (p < 0.05) with an R2 of 0.35.

Variable	Coefficient	Standard error	Unadjusted OR	95% CI lower	95% CI upper	p-value	Adjusted OR	95% CI lower	95% CI upper	p-value
Age	-0.397	0.458	0.942	0.567	1.563	0.816	0.672	0.274	1.650	0.386
Height	0.848	0.540	1.447	0.851	2.459	0.172	2.335	0.811	6.727	0.116
Excess body water	0.733	0.467	1.120	0.674	1.860	0.66	2.082	0.834	5.198	0.116
Serum osmolality	-0.412	0.414	0.641	0.369	1.113	0.113	0.662	0.294	1.491	0.320
Eosinophil	0.099	0.360	0.922	0.555	1.534	0.755	1.104	0.545	2.238	0.783
Serum albumin	0.189	0.396	1.093	0.658	1.816	0.732	1.208	0.555	2.627	0.634
Serum creatinine	1.211	0.441	2.425	1.305	4.505	0.005	3.358	1.415	7.969	0.006
Serum calcium	-0.225	0.396	0.903	0.543	1.502	0.693	0.798	0.368	1.733	0.569
Serum sodium	-0.431	0.359	0.868	0.522	1.444	0.584	0.650	0.321	1.313	0.230
Hematocrit	-0.691	0.466	0.925	0.557	1.536	0.762	0.501	0.201	1.249	0.138
Arterial pH	-0.418	0.345	0.886	0.531	1.477	0.641	0.659	0.335	1.294	0.226

**Figure 2:**
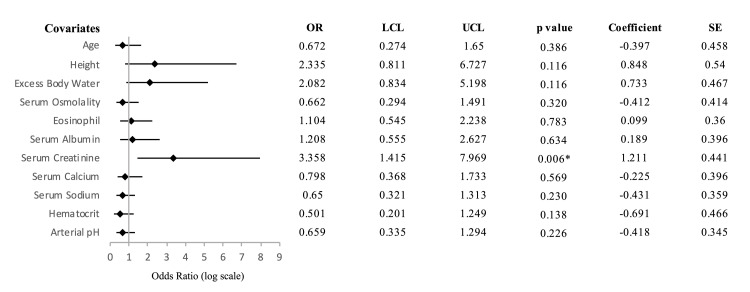
Forest plot of multivariate model showing covariates associated with sinonasal polyposis. The odds ratios and 95% confidence intervals are displayed with covariates where confidence intervals do not cross an odds ratio of 1, signifying statistical significance. * P < 0.05 indicates a statistically significant association. OR: odds ratio; LCL: lower 95% confidence interval limit; UCL: upper 95% confidence interval limit; SE: standard error.

Multivariate logistic regression analysis used all the variables to control for multiple confounding factors. After VIF analysis, sex, weight, and TBW (VIF = 42.11, 76.29, and 116.54, respectively) were excluded from the model, which made it more reliable and stable. The likelihood of developing sinonasal polyposis was twice as high in patients with excess body water compared to those without, as suggested by an adjusted OR of 2.08. However, this association was not statistically significant (95% CI: 0.83-5.19, p = 0.116). The regression model revealed a weaker trend for other parameters except serum creatinine. Following stepwise backward elimination, the final model identified serum creatinine level as the only significant predictor of sinonasal polyposis, with a strong association indicated by a p-value of 0.006 and a 95% CI of 1.42-7.97. The VIF for multicollinearity suggested that hydration status or related factors did not confound the relationship between serum creatinine and sinonasal polyposis. The OR indicates that for every standard deviation increase in serum creatinine level, the odds of having sinonasal polyposis increased by approximately 3.4 times. The coefficient for serum creatinine (log-odds coefficient = 1.21) indicates that for every one-unit increase in serum creatinine, the log odds of having sinonasal polyposis increased by 1.21 (Figure 2).

**Figure 3:**
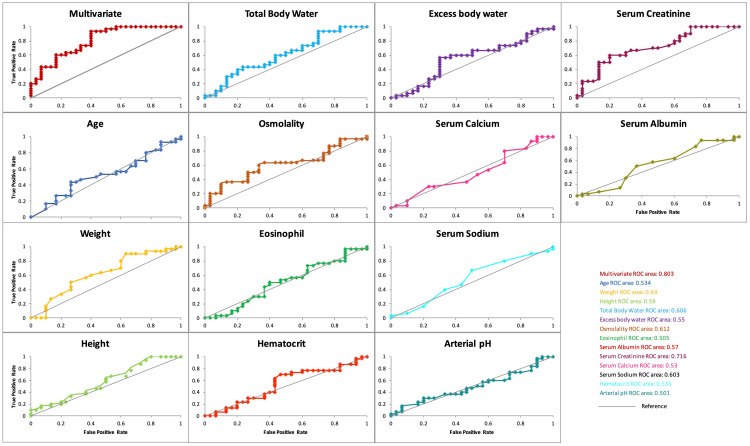
Receiver operating characteristic (ROC) curve for predicting outcomes. The final multivariate model had the maximum area under the curve (AUC) with good predictive ability (0.803, p = 0.03), followed by serum creatinine with fair predictive ability (0.716, p = 0.001).

Though the final multivariate model had only serum creatinine as a significant predictor, incorporating additional clinical and biochemical parameters, including excess body water, resulted in an improved AUC of 0.803, indicating superior discriminative capability compared to univariate models. The model correctly distinguished between patients with and without polyposis approximately 80.3% of the time compared to the univariate model for serum creatinine alone, which had an AUC of 0.716, suggesting fair discriminative ability. The remaining univariate models had AUC values below 0.7 and were not statistically significant (p > 0.05). The final model’s accuracy was 66.7%, with a sensitivity of 73.3% and a specificity of 60%, which means that the model correctly identified 73.3% of the patients with sinonasal polyposis and 60% without the condition. Thus, the model is more effective in identifying true-positive polyposis cases than correctly identifying true-negative cases. The model's overall significance (p < 0.05) supports its validity in predicting the outcomes (Figure 3).

## Discussion

This study investigated the impact of body fluid load on the presence of sinonasal polyps. We noted elevated total and excess body water levels in affected patients compared to controls. Although there was a positive correlation between excess body water and the development of sinonasal polyps, the evidence supporting this relationship was limited. Notably, serum creatinine levels emerged as a robust predictor of sinonasal polyposis. However, secondary biochemical parameters, such as serum albumin, serum calcium, hematocrits, serum sodium, arterial pH, and eosinophil count, exhibited only weaker trends. Covariates, including age, sex, weight, and height, were considered in the analysis to mitigate their confounding effects.

The higher mean body water levels (total and excess) in CRSwNP than in controls in our study suggest that body fluid levels might influence the development of sinonasal polyposis polyp formation. However, while a positive trend exists between excess body water and the disease suggested by an OR of 2.08, the evidence is weak (p = 0.116) to confirm its direct impact on polyp formation. The excess body water levels, combined with serum creatinine and other biochemical markers like serum osmolality, may have some predictive role in sinonasal polyposis, as indicated by the multivariate model's high AUC (0.803). Studies to determine if sinonasal polyps are caused by abnormal water flow through the sinonasal mucosa have been previously performed [12]. However, no comprehensive studies evaluate the direct relationship of body water levels with disease occurrence to support these findings. Initial theories proposed that polyps could develop from adenomas, fibromas, mucosal exudates, cystic expansions of excretory ducts, or glandular or lymphatic obstruction, causing tissue swelling [5,13]. Additional theories suggest that the underlying factors may be recurrent infections that cause lymphangitis or glandular hyperplasia. Delgado-Dolset et al. proposed a model in which epithelial rupture and necrosis lead to protrusions from the lamina propria, followed by subsequent epithelial repair. Many of these early hypotheses aimed to elucidate the mechanistic processes involved in polyp formation [14].

We also explored other potential biochemical markers as part of our secondary objective. Our second finding was a statistically significant difference in serum creatinine levels between cases and controls, with patients diagnosed with sinonasal polyposis exhibiting higher serum creatinine levels (0.94 mg/dL) compared to those without the condition (0.80 mg/dL). This suggests a potential association between elevated serum creatinine levels and sinonasal polyposis. Regression analysis further supported the significance of serum creatinine level as an independent and positive predictor of sinonasal polyposis. Thus, serum creatinine, commonly used as a renal function marker, could indicate the underlying metabolic or inflammatory processes contributing to nasal polyp development. Recent studies have proposed a potential connection between chronic kidney disease (CKD) and several sinonasal issues, such as nosebleeds (epistaxis), infections, and olfactory dysfunction [15]. However, studies investigating the association between CKD and CRS remain sparse. Although some case reports have addressed sinusitis concerning kidney disease, comprehensive studies on this topic are limited [16,17]. The relationship between CKD and CRS remains poorly understood and is still under investigation. Future studies to understand potential shared pathways (such as systemic inflammation, immune response, tissue remodeling, or oxidative stress) must establish whether this correlation translates into clinical implications. Notably, the fact that the creatinine levels in our study were significantly elevated in patients with sinonasal polyposis but still within the normal range suggests that, while there is a statistical difference, it does not imply that creatinine levels in patients with sinonasal polyposis are pathologically elevated, as one might expect in conditions such as end-stage renal disease. This finding supports the idea that the association between creatinine and sinonasal polyposis may reflect a more subtle or elusive physiological process rather than a direct consequence of impaired renal function.

Additionally, our study identified an inverse relationship between serum osmolality and the occurrence of sinonasal polyposis, suggesting that increased osmolality might reduce the likelihood of polyp formation. However, its clinical impact may be minimal, as reflected by a p-value of 0.119. Previous studies have suggested hyperosmolarity can activate nasal cells and increase inflammatory mediators, typically associated with worsening nasal conditions [12]. The discrepancy may indicate that different mechanisms are at play in polyp formation or that certain regulatory factors mitigate the effects of increased osmolality in the nasal environment.

Research indicates that chronic eosinophilic inflammation in nasal polyps is maintained by an internal mechanism rather than persistent exposure to external allergens. This self-sustaining process is driven by the local release of inflammatory mediators within the nasal mucosa, which promotes the development and enlargement of nasal polyps, causes tissue edema, and attracts additional inflammatory cells. Although eosinophils are critical players in this process, mast cells also play a significant role in intensifying the inflammatory response [18,19]. While eosinophils and mast cells are key to inflammatory response, our study found no significant impact of blood eosinophilia on the odds of having CRSwNP, and eosinophil levels were comparable between cases and controls. This aligns with findings by Lou et al. [20] that blood eosinophilia may not accurately reflect tissue eosinophilia, limiting its usefulness as a predictive marker in such conditions. Furthermore, although a previous study by Machado et al. [7] noted differences in serum albumin levels between patients with nasal polyposis and controls, our study's slight differences were not statistically significant.

This study had several strengths and certain limitations. As one of the first studies to explore body fluid dynamics in sinonasal polyposis among CRSwNP patients, it opens up a novel area of CRS research. It provides a comprehensive perspective on the potential factors in nasal polyp development by measuring diverse physiological parameters, including TBW, serum osmolality, creatinine, and eosinophil count. Using standardized formulas such as Watson’s formula for TBW enhances methodological rigor. However, the study’s primary objective, assessing the link between TBW and sinonasal polyposis, yielded no statistically significant results, likely due to the limited sample size. Future studies with larger sample sizes are recommended to provide a more definitive assessment of this association. The absence of similar studies in the literature also constrains comparative analysis, making it challenging to contextualize our findings within the existing body of research. Additionally, while we observed a significant association between serum creatinine level and sinonasal polyposis, the observational design precludes causal inference.

## Conclusions

In our investigation into the role of total body fluid dynamics in sinonasal polyposis, we observed elevated levels of total and excess body water in affected patients compared to controls. The regression model indicated a positive correlation between excess body water and the development of polyposis, with weaker trends for other parameters. However, given the limited evidence, there may not be a direct association between body water levels and the presence of nasal polyps, and further research involving larger sample sizes across diverse geographic regions is recommended to validate and clarify these relationships. Additionally, our study revealed a notable increase in serum creatinine levels in CRSwNP patients, suggesting a potential connection between kidney function and nasal polyps, which merits further exploration of renal factors in CRS. Overall, this study underscores the complexities of CRSwNP and the necessity for ongoing research to fully elucidate the potential associations between body fluid dynamics and sinonasal polyposis.
